# AM281, Cannabinoid Antagonist/Inverse agonist, Ameliorates Scopolamine-Induced Cognitive Deficit

**Published:** 2012

**Authors:** Mohammed Rabbani, Golnaz Vaseghi, Valiollah Hajhashemi

**Affiliations:** 1 *Department of Pharmacology, Isfahan Pharmaceutical Sciences Research Centre, School of Pharmacy and Pharmaceutical Sciences, Isfahan University of Medical Sciences, Isfahan, Iran*

**Keywords:** AM281, Cannabinoid antagonist, Cognitive deficit, Mice, Scopolamine

## Abstract

**Objective(s):**

Cannabinoids have been implicated in memory deficit. We examined the effect of AM281, cannabinoid antagonist/inverse agonist in prevention of scopolamine-induced cognitive deficit.

**Materials and Methods:**

Object recognition task was used to evaluate memory in mice. Exploration time in the first and the second trial was recorded. The differences in exploration between a previously seen object and a novel object in second trial were taken as an index of memory. Scopolamine and AM281 were administrated at the same time, 40 min before second trial in the treatment group.

**Results:**

Object discrimination was impaired after scopolamine (2 mg/kg; IP) administration. AM281 (2.5, 5 mg/kg; IP) significantly restored object recognition ability in mice treated with scopolamine by 75%.

**Conclusion:**

This study extends earlier findings, suggesting the interaction of cannabinoid and cholinergic system in memory. Additionally cannabinoid antagonists seem to show variable pharmacological properties.

## Introduction

Based on “cholinergic hypothesis of geriatric and Alzheimer disease (AD) memory dysfunction” antimuscarinc drugs have been widely used as amnesic drugs in animal to mimic the cognitive dysfunction observed in dementia and AD (1). Scopolamine, a competitive antagonist of muscarinic acetylcholine receptors has been extensively used in related studies to disrupt performance on several reference memory tasks, such as object discrimination, radial arm maze, water maze and object recognition (2). Reversal of scopolamine-induced memory deficit has been used as an initial screening method for identification of therapeutic candidates for cognitive disorders.

Cannabinoid agonists such as Δ^9^–tetrahydrocannabinol (THC), anandamide, and WIN 55,212-2 (WIN-2) have long been known to impair memory in humans and laboratory animals (3). Blockade of cannabinoid receptors (CB1) by SR141716A was proved to enhance memory acquisition in various test paradigms such as social recognition memory task (4), radial maze spatial memory task (5), inhibitory avoidance (6) and elevated T-maze (7). The status of CB1 receptor antagonist in relation to their memory enhancement properties is not quite consistent. For example, SLV330 (cannabinoid antagonist) failed to improve recognition in object recognition test (8). AM281, CB1 receptor antagonist/inverse agonist is another compound that has not been evaluated for its effect on memory. However, in locomotor activity measurement in mice, AM281 at a dose of 0.3 mg/kg increased the spontaneous locomotor activity. SR141716A produced similar effect on locomotor activity, but only at much higher doses of 20 mg/kg. Therefore, it appears that CB1 receptor antagonists/inverse agonists bear different pharmacological properties when they are used in various behavioral paradigms (9).

The aim of the present study was to evaluate the action of AM281 on scopolamine-induced memory deficit using object recognition paradigm. 

## Materials and Methods

Animals- Male NMRI mice (8-12 weeks, Pasteur Institute, Tehran, Iran) with average weight of 20-30 g were used; they were housed in the experiment place one day before study. All the studies were performed between 8-12 AM. At least 6 mice were used in each group.

Drugs- Scopolamine sulphate (Daru Pakhsh, Iran) was diluted with normal saline 0.9% and AM281 (Sigma, USA) was dissolved in normal saline 0.9% and DMSO (4% v/v).

Drug treatments- The following groups were included: Scopolamine (2 mg/kg) +DMSO vehicle; AM281 2.5, 5 mg/kg + saline; scopolamine + AM281 (2.5, 5 mg/kg); saline +DMSO vehicle. All drugs were administrated 40 min before T2. 

**Figure 1:**
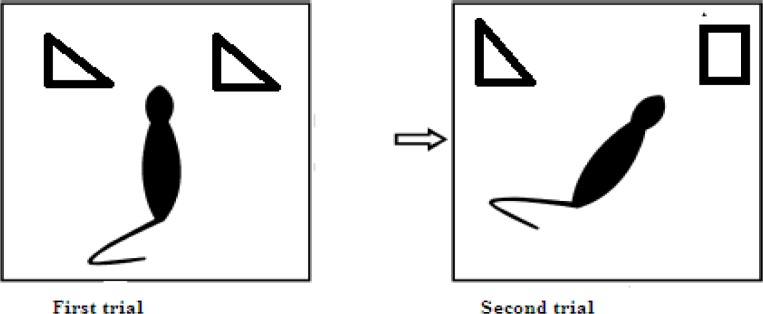
Schematic representation of memory performance

Object recognition task- Novel object recognition (NOR) test is a sensitive procedure for evaluating compounds effect on cognition activity (10). The apparatus consisted of a wooden cage (35×35×40 cm) with the black walls and white floor which was located in the noise free room. The day before the test, each animal was allowed to explore the apparatus for 15 min. On the test day, animals were given the opportunity to explore two identical objects for a 20 sec in 12 min cut-off time (T1). After 15 min, two objects were presented to be explored by animal, one of which was the same as the one in the first exploration trial, and the other one was a new object. Objects are logos different in shapes and colors. Animals would explore the arena for 5 min and the total time spent in exploration of the familiar (F) and the new object (N) was measured. Recognition index (RI) was defined using the following formula: (N-F/N+F) % (10). All the procedures were recorded using a camera placed above the experimental apparatus (Figure 1).

Statistical analysis- T1 was recorded for each mouse, RI was calculated, and the data were analyzed using one way ANOVA followed by *post-hoc* analysis, *P*< 0.05 was considered as significant. Results were expressed as mean±SEM. SigmaStat software (Systat corporation) was used for all statistical analyses.

**Figure 2:**
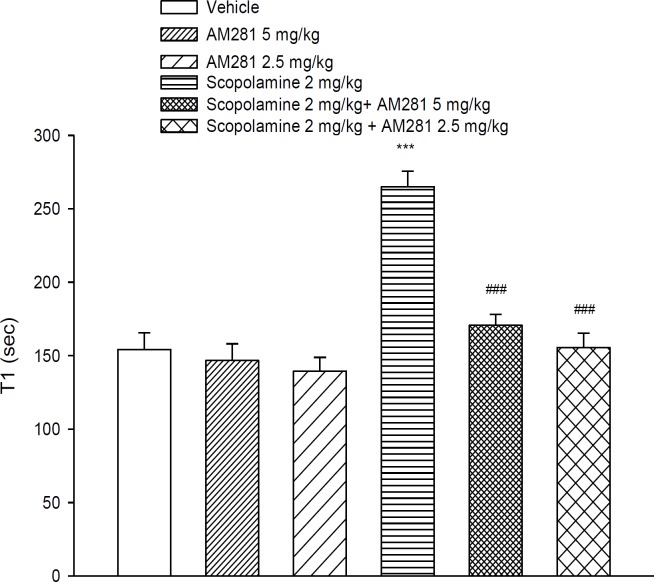
Time required to explore 20 sec. of object exploration on trial 1 (duration of T1), compared with control values in the object recognition tasks.

Results are expressed as mean ±SEM. ***P< 0.001 compared with vehicle and AM281 (2.5, 5 mg/kg) alone values,

^###^ *P*< 0.001 compared with scopolamine values (n= 6 in each group)

**Figure 3:**
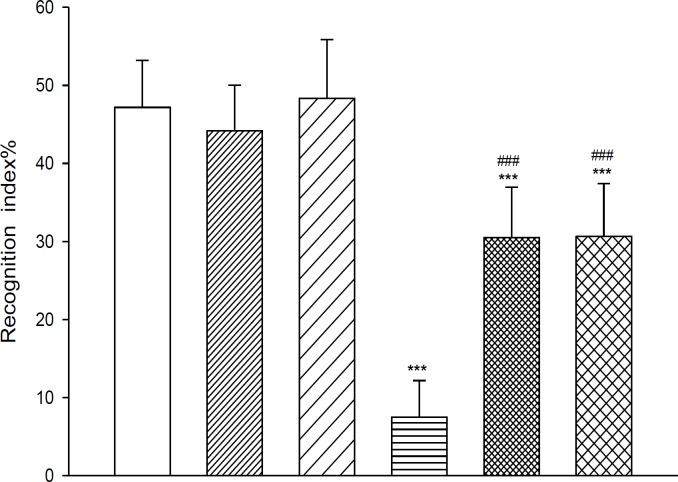
Effect of different doses of AM281, cannabinoid antagonist, on memory performance (expressed as recognition index) disrupted by scopolamine, in the object recognition tasks

Results are expressed as mean±SEM ***P< 0.001 compared with vehicle and AM281 (2.5, 5 mg/kg) alone value, ^###^*P *< 0.001 compared with scopolamine values (n= 6 in each group)

## Results

***Effect of scopolamine*** ***on memory performance***

During the first trial (Figure 2) T1 significantly increased by scopolamine (*P*< 0.01). Figure 3 shows the recognition index in the second trial of the object recognition task after scopolamine. The RI score decreased in the scopolamine condition compared to the saline conditions, as shown by the *post-hoc* analysis (*P*< 0.001). 

***Effect of*** ***AM281 on memory performance***

AM281 did not significantly affect T1 and RI exploration scores in comparison with vehicle group. Figure 2 and 3 illustrate the T1 and RI score after AM281 treatments. 

***AM281 and scopolamine co- treatment***

Administration of AM281 with scopolamine significantly decreased exploration time (T1) in comparison with scopolamine alone (*P*< 0.001).

RI scores after co-treatments can be seen in Figure 3. A significant treatment effect was present (*P*< 0.001). *Post-hoc* analysis revealed that the RI score was increased after scopolamine co-treatment with AM281 compared to the scopolamine but the performance did not return to the normal value (*P*< 0.001).

## Discussion

The effects of AM281 were evaluated on scopolamine disrupted cognitive deficit using object recognition paradigm. The data suggested that AM281 could reverse the cognitive deficit.

The object recognition task allows rapid evaluation of memory performance in mice and rats (11). In this method no rewarding or aversive stimulation is used during training, the learning occurs under normal condition and relatively low stress or arousal (12). The effect of scopolamine on the mice performance was examined with a single injection of 2 mg/kg 10 min before T1 which caused amnesia.

As it is shown in Figure 2 and 3, at the first and second trial, T1 was high and RI was very low in scopolamine injected animals. These animals could not discriminate between the new object and the familiar one, therefore recognition was significantly deficit. Using the object recognition task, AM281 by itself did not have any effect on memory performance in mice but co-treatment with scopolamine partially improved the cognitive deficit.

Despite its structural difference with other extensively studied inverse agonists, such as rimonabant, AM281 behaves as an inverse agonist at CB1 receptor *in vitro*. Showing relevant *in vitro* potency and selectivity profile, it seemed to be a good candidate for memory enhancement, but further preclinical studies are needed in animal models of cognitive dysfunction. In addition, these data are consistent with earlier published results suggesting that CB1 receptor knocked out mice show an improved object recognition performance compared with wild-type mice (13).

The improvement of cognitive deficit is thought to be due to AM281 action at the level of endocannabinoid receptors. Previous study had reported that SLV330 alone could not improve cognitive deficit; however the combination of SLV330 and donepezil improved the cognition (8). MK-7128 (another cannabinoid antagonist) completely improved scopolamine-induced learning and memory deficits in mice (14). The differences between these results could reflect the sensitivity of endocannabinoid and the degree of interaction between cholinergic, endocannabinoids or perhaps because of its inverse agonistic character. AM281 could increase the release of acetylcholine in hippocampus slices (15). The reversal of scopolamine- induced cognitive deficit may occur via targets other than CB1 receptor (9). Finally, although our data indicats that AM281 improves the recognition memory performance, additional studies are needed to investigate the performance improvement in other recognition memory models such as Y-maze to define the role of CB1R in recognition deficit.

## Conclusions

AM281 improved memory impairment after administration of scopolamine and it reversed memory performance but not to the normal value. Results of these experiments showed that although cannabinoid antagonists improve memory, each of them affect memory in a different way. Future studies may help to address the issues of pharmacological character of different CB1 receptor ligands.
